# Analysis of Zika virus neutralizing antibodies in normal healthy Thais

**DOI:** 10.1038/s41598-018-35643-6

**Published:** 2018-11-21

**Authors:** Wannapa Sornjai, Janejira Jaratsittisin, Prasert Auewarakul, Nitwara Wikan, Duncan R. Smith

**Affiliations:** 1 Department of Microbiology, Faculty of Medicine, Siriraj Hospital, Mahidol University, Bangkok, Thailand; 2 Institute of Molecular Biosciences, Mahidol University, Bangkok, Thailand

## Abstract

Zika virus (ZIKV) infections have been reported from all over Thailand, but the number of reported cases remains low, suggesting a degree of immune protection against ZIKV infection. To address this possibility, the presence of ZIKV neutralizing antibodies was determined in serum from 135 healthy Thai adults with a plaque reduction neutralization test (PRNT), and a number of samples were subsequently analyzed for the presence of neutralizing antibodies to dengue virus (DENV) and Japanese encephalitis virus (JEV). Results showed that 70.4% (PRNT_50_ ≥ 10), 55.6 (PRNT_50_ ≥ 20) or 22.2% (PRNT_90_ ≥ 20) of the samples showed neutralizing antibodies to ZIKV. Detailed analysis showed no association between the presence of neutralizing antibodies to other flaviviruses (DENV, JEV) and the presence of ZIKV neutralizing antibodies. These results suggest that the level of ZIKV neutralizing antibodies in the Thai population is enough to dampen the transmission of the virus in Thailand.

## Introduction

ZIKV was first isolated from a sentinel monkey in Zika forest Uganda in 1947^1^, and the virus was isolated from *Aedes africanus* mosquitoes in the same forest a year later^1^. The first reported human case of ZIKV infection was reported nearly a decade later, with infection again occurring in Zika forest in Uganda^2,3^. Between the initial identification of ZIKV and 2007 only a few sporadic cases of human ZIKV infection in Africa and Asia were reported (as reviewed elsewhere^4^). In 2007 a small outbreak on the islands of Yap State in Micronesia represented the first time the virus was detected outside of Africa and Asia and analysis suggested this virus has originated in Southeast Asia^5^. The virus again emerged from Southeast Asia in 2013 where it was detected as the causative agent in an outbreak of Zika fever in French Polynesia^6^. The virus subsequently spread to many of the islands in the Pacific Ocean, and was detected in Brazil in March 2015^7,8^, and within one year more than a million cases of infection were reported. From Brazil the virus spread quickly to other countries in South, Central and North America (as reviewed elsewhere^4^).

While serological studies have suggested the presence of ZIKV in Southeast Asia for more than 60 years^9–11^, definitive evidence of the presence of the virus was first reported in 1966^12^, and the first virologically confirmed case of human infection was reported from Cambodia in 2010^13^. In 2013 two tourists to Thailand were diagnosed on their return to their home countries with ZIKV infection^14,15^ and a subsequent retrospective study by the Thai Ministry of Health confirmed that the virus was present in Thailand and causing disease in the local population^16^, with the earliest confirmed cases dating to infections occurring in 2012. Currently, some 1,600 cases of ZIKV infection are reported to have occurred in 2016 and 2017 by the Thai Ministry of Public Health^17^. The basis of the markedly different population impact of ZIKV in the Americas and Southeast Asia remains unknown.

At least three human pathogenic mosquito transmitted flavivirus circulate in Thailand, and in addition to ZIKV, both dengue virus (DENV) and Japanese encephalitis virus (JEV) circulate. DENV consists of four closely related, but antigenically distinct viruses^18^, termed DENV 1 to 4 all of which are endemic in Thailand^19^. Currently the main public health problem caused by these viruses is infections with DENV, as there are an average of 50,000 cases per year in Thailand, and DENV infection is the leading cause of hospitalization amongst children in Southeast Asia^20^. There is a national vaccination program to protect against JEV infections established in 1990^21^, however, JEV infection was the leading cause of hospitalizations for encephalitis in a study undertaken between 2003 and 2005^22^.

DENV infection induces protective immunity that lasts for many decades and possibly lifelong^23^. However the immunity raised is against the homotypic virus only, and only limited or transient protection is generated against heterotypic DENV, and thus multiple infections with DENV are possible^24^. Studies have shown that some 90% of Thai adults have antibodies against at least one DENV^25–27^. Given the national JEV vaccination campaign, adults and children below the age of around 30 are likely to have protective antibodies against JEV, while those older than that may not. The flaviviruses show high levels of homology^28^, including in the main antigenic determinant the envelope (E) protein^29^, and thus, as part of the immune response to an infection, cross reactive antibodies are raised^30^. While some studies have shown that anti-DENV antibodies can neutralize ZIKV^31,32^, other studies have shown that anti-DENV antibodies may enhance ZIKV infection through the process of antibody dependent enhancement (ADE) of infection^33,34^. Mouse studies have suggested that protection or enhancement in ZIKV infection may depend upon the level of anti-DENV antibodies present^35^. However, non-human primate studies have suggested that preexisting immunity to DENV has no effect on ZIKV infection^36,37^, and recent longitudinal studies on populations from Asia and the Americas have suggested that only low levels of cross neutralization of ZIKV are generated by prior DENV infection^38^. Nevertheless, it remains possible that the different population impact of ZIKV seen in Southeast Asia as compared to the Americas is a consequence of the existence of cross protection as a consequence of prior flaviviral infections and/or vaccination. This study sought to investigate the presence of ZIKV neutralizing antibodies in normal healthy Thais. In this respect the study is unique from other studies investigating ZIKV neutralizing antibodies that have mainly focused on the presence of these antibodies in patients with recent infections^39–42^.

## Materials and Methods

### Blood sample collection and preparation

The study was performed in accordance with the Helsinki Declaration and was conducted after approval by the Mahidol University Central Institutional Review Board (Number: COA No. MU-CIRB 2017/067.2404). Written informed consent was received from all participants (cohort and controls). A total of 10 ml peripheral blood was collected into clot blood tubes (Greiner Bio One (Thailand) Ltd., Chonburi, Thailand) from the cohort of 135 healthy volunteers recruited through advertisement of the project at the Institute of Molecular Biosciences, Mahidol University during April–December 2017. There were 24 males and 111 females, with an age range of 18–63 years old. Whole blood samples were placed at room temperature for 30 min. Two additional serum samples, one known ZIKV infection positive and one known flavivirus (DENV/JEV/ZIKV) negative were used as control samples. Serum was separated by centrifugation at 3,000 rpm for 10 min and kept at −20 °C until required.

### Cells and viruses

Vero (ATCC Cat No. CCL-81) and LLC-MK_2_ (ATCC Cat No. CCL-7) cells were grown in Dulbecco’s modified Eagle’s medium (DMEM, Gibco, Invitrogen, Carlsbad, CA) supplemented with 5% heat inactivated fetal bovine serum (FBS; Gibco, Invitrogen) and 100 units/mL of penicillin/streptomycin (Gibco, Invitrogen). Cells were incubated at 37 °C with 5% CO_2_. All viruses used in this study are shown in Supplemental Table S1. These viruses were propagated in C6/36 cells (ATCC Cat No. CRL-1660) with Minimum Essential Media (MEM; Gibco) supplemented with 10% FBS and 100 units/mL of penicillin/streptomycin. Virus supernatants were partially purified by centrifugation and supplemented with FBS up to 20%. All viruses were kept at −80 °C until required and their titers were quantitated on Vero cells or LLC-MK_2_ cells by the standard plaque assay as previously described^43^.

### Plaque Reduction Neutralization Test (PRNT)

Plaque reduction neutralization test (PRNT) of ZIKV and JEV was determined on Vero cells while PRNT of all DENV serotypes were determined on LLC-MK_2_ cells. Cells were seeded into 6- well plates at a density of 6 × 10^5^ cells/well and grown at 37 °C for 24 h. Human serum was heat inactivated at 56 °C for 30 min. To detect neutralizing antibodies against ZIKV, all inactivated serum samples were initially diluted with BA-1 medium containing Medium 199 (Gibco, Invitrogen) and 10 mg/mL of bovine serum albumin fraction V at a dilution of 1:5 or 1:10 followed with serial two-fold dilutions. Some samples were diluted until the endpoint of neutralization activity was reached. Viruses were diluted with BA-1 medium to obtain a virus infectivity concentration of 600–2,000 PFU/mL (dependent upon virus). After that, 100 μl of prepared virus was incubated with an equal volume of each diluted serum or serum free BA-1 medium (non-neutralization control) in duplicate at 37 °C for 1 h. The virus and human serum mixture was inoculated onto confluent Vero or LLC-MK_2_ cells and incubated at 37 °C with constant agitation for 2 h. Inoculated cells were gently overlaid with first overlay solution containing 2X nutrient and 0.6% Seakem LE agarose (Cambrex, Bio Science Rockland Inc., Rockland, ME) for ZIKV or 1.6% for other viruses and maintained at 37 °C with 5% CO_2_. On day six of incubation, cells were gently overlaid with a second overlay solution containing 2X nutrient, 0.5% neutral red and 1.6% Seakem LE agarose and incubated further for 15–17 h. Plaque number was counted and PRNT_50_ and PRNT_90_ are defined as a reciprocal of the highest dilution of tested serum that resulted in a reduction of viral infectivity by 50% or 90% when compared to the non-neutralization control, respectively. All PRNT results generated can be found in the supplemental data file.

### Statistical analysis

The difference between flavivirus neutralizing antibody profiles in ZIKV PRNT_90_ < 20 and ZIKV PRNT_90_ ≥ 20 cohorts was compared by independent sample *t*-test. A *p*-value of <0.05 was considered significant. This analysis was performed using PASW statistics 18 (SPSS Inc., Chicago, IL).

## Results and Discussion

### Zika virus neutralizing antibodies in healthy Thai citizens

In a previous study, serum from 21 acute undifferentiated fever patients collected in Thailand was examined for immunoreactivity to ZIKV, DENV, JEV and CHIKV envelope proteins^44^. Immunoreactivity to at least one of the antigens was found in 20 of the 21 samples, with two samples showing immunoreactivity only to ZIKV E protein. However, a total of 14 of the samples showed immunoreactivity to ZIKV and at least one other antigen. This would suggest that ZIKV neutralizing antibodies are present at between approximately 10% (2/21) and 76% (16/21) of the Thai population. For this reason a cohort size of 135 was selected which would be expected to have between 10 and 100 samples with neutralizing antibodies. Therefore to study the presence of ZIKV neutralizing antibodies in healthy Thai adults, serum was collected from 135 healthy volunteers. The cohort was aged 18–63 years of age, consisting of 24 Thai males and 111 Thai females. Neutralizing antibodies were detected and quantitated by the gold standard for determining flavivirus neutralizing antibodies, the plaque reduction neutralization test (PRNT). PRNT of all human serum samples was initially determined against ZIKV strain SV0010/15 which was isolated from a Thai Zika fever patient^16^ and represents the ZIKV currently circulating in Thailand. In an initial analysis, all samples were screened at a low level of stringency with ZIKV antibody positive samples being defined as those showing a PRNT_50_ ≥ 10. This cut-off value has been used in several recent studies determining levels of ZIKV neutralizing antibodies^39,45,46^, as shown in Supplemental Table S2, and is additionally the default level recommended by the WHO in PRNT for DENV^47^. In this preliminary screen, samples were not subjected to assay of the endpoint of neutralization and only three conditions were screened (control, 10 fold dilution and 20 fold dilution). A total of 95 out of 135 samples (70.4%) showed PRNT_50_ ≥ 10 to ZIKV strain SV0010/15 (see Table 1 and Supplemental Table S3), while 73 out of the 135 samples (54.1%) showed a PRNT_50_ ≥ 20 (see Table 1 and Supplemental Table S3).

**Table 1:** Summary of plaque reduction neutralization test of 135 human serum samples against ZIKV strain SV0010/15.

PRNT_50_ value	Number of cases	Percentage (%)
<10	40	29.6
10	22	16.3
≥20	73	54.1
Total	135	100

However, the WHO recommendation for DENV PRNT analysis suggest that in flavivirus endemic areas a PRNT_90_ cutoff value is preferred^47^, and several recent studies have used a 90% reduction in virus titer as the criteria for PRNT of ZIKV antibodies^40–42,48^. However, these studies have variously used a 10 fold^40,42^ or a 20 fold^41,48^ initial dilution (see Supplemental Table S2). For greater stringency, this study used a 20 fold initial dilution, and thus positivity was defined as PRNT_90_ ≥ 20, and samples were further diluted to determine the endpoint of neutralization activity. At this level of analysis a total of 30 samples (22.2%) were positive for ZIKV neutralizing antibodies. (See Supplemental Table S4). We additionally recalculated the samples to find the number of samples that would be positive with a 50% reduction of titer, and a total of 75 samples (55.6%) were positive for ZIKV antibodies at PRNT_50_ ≥ 20 (see Supplemental Table S5). This analysis was undertaken independently from the first screening analysis (which showed a PRNT_50_ ≥ 20 of 54.1%).

### DENV, ZIKV and JEV neutralizing antibodies in healthy Thai citizens

It has been clearly established that anti-DENV antibodies can cross react with, and neutralize ZIKV^31,32^. For this reason, the neutralizing antibody profile to four laboratory adapted DENV serotypes (DENV 1–4 lab), four clinically isolated DENV serotypes (DENV 1–4 DF) as described previously^49^ and JEV was evaluated for all 30 ZIKV neutralizing antibody positive samples (as defined by PRNT_90_ ≥ 20), as well as a roughly sex and age matched equal number of ZIKV neutralizing antibody negative samples (Supplemental Tables S6 and S7). Samples were diluted to determine the endpoint of neutralization activity, and value of PRNT_90_ ≥ 20 was defined as a positive result for all viruses. To confirm the validity of the PRNT assay, two further samples were screened. These were a 57 year old Caucasian resident of Thailand with no history of febrile illness, and a 22 year old female Thai with recent, clinically confirmed ZIKV infection. The Caucasian sample showed no evidence of neutralization for ZIKV, JEV or DENV, while the Thai sample showed a PRNT_90_ ≥ 20 titer of 1,280 as well as neutralizing antibodies to JEV and DENV (Supplemental Table S8).

For the ZIKV neutralizing antibody positive samples (Table 2) two serum samples (S9 and S33) exhibited high levels of neutralizing antibodies to ZIKV but not to any of the DENVs or JEV. This result strongly suggests that these two serum samples carry specific ZIKV neutralizing antibodies, raised as a consequence of ZIKV infection. Two further serum samples (S89 and S103) possessed neutralizing antibodies to ZIKV and JEV, but showed no evidence of DENV neutralizing antibodies, suggestive of ZIKV infection and JEV vaccination and/or infection. In Thailand, a mouse brain derived-inactivated JEV vaccine was initially included into the routine immunization schedule in 1990^21^ and it became a nationwide vaccination in 2000. Both S89 and S103 were 29 years of age at time of sample donation, and as such the JEV vaccination was possibly administered as part of the vaccination program, however detailed vaccination history of all participants was not collected. However, despite the presence of a national JEV vaccine program, cases of JEV encephalitis have been reported, even in vaccinated cases^22^, so natural JEV infection cannot be ruled out. The rest of the samples showed complex profiles, with evidence of neutralizing antibodies to multiple DENV serotypes and/or JEV. Markedly however, the PRNT profiles for the ZIKV neutralizing antibody negative samples (Supplemental Table S9) were equally as complex. Markedly, if ZIKV neutralization was occurring as a consequence of cross-neutralizing antibodies, it would be expected that there would be a relationship between the degree of other flaviviral exposure and ZIKV neutralization. However there was no association between ZIKV neutralizing antibody positivity and other flaviviral exposure, when DENV was treated as 4 flaviviral exposures (Supplemental Table S10) (*p* value = 0.102) or as one (Supplemental Table 11) (*p* value = 0.286) based on independent sample *t*-test. This suggests that where high titers of ZIKV neutralizing antibodies are found, they have arisen through past ZIKV infection, and as such would be consistent with the study by Montoya and colleagues^38^.

**Table 2:** Full screen of neutralizing antibody titer for DENV (1–4) and JEV by plaque reduction neutralization test of ZIKV PRNT_90_ ≥ 20 positive serum samples.

Sample	Gender	Age	ZIKV SV0010/15	JEV	DENV 1 lab	DENV 1 DF	DENV 2 lab	DENV 2 DF	DENV 3 lab	DENV 3 DF	DENV 4 lab	DENV 4 DF
S9	F	25	160	<20	<20	<20	<20	<20	<20	<20	<20	<20
S33	M	24	1,280	<20	<20	<20	<20	<20	<20	<20	<20	<20
S36	F	56	80	160	640	640	80	80	320	160	20	40
S39	F	47	320	<20	160	160	320	640	80	20	20	40
S40	F	53	160	80	80	80	20	20	40	20	<20	20
S41	F	50	160	80	160	160	80	20	40	80	<20	20
S43	F	49	20	320	320	640	320	320	160	80	80	160
S50	F	24	640	320	40	20	20	20	40	<20	40	80
S57	F	23	640	320	40	80	320	320	40	<20	<20	20
S62	F	37	160	<20	160	160	20	20	20	20	<20	20
S68	F	37	160	40	<20	20	<20	<20	<20	<20	40	20
S70	F	52	40	80	160	320	160	160	80	40	40	40
S75	M	37	80	<20	640	320	320	320	320	320	20	40
S76	F	36	160	<20	20	80	40	40	20	20	<20	<20
S81	F	52	320	<20	80	160	160	160	80	80	<20	20
S82	F	37	80	<20	80	80	40	40	40	20	<20	<20
S84	F	25	20	320	40	40	1,280	1,280	40	80	<20	20
S86	M	40	80	20	80	80	80	40	<20	<20	<20	<20
S89	F	29	160	160	<20	<20	<20	<20	<20	<20	<20	<20
S92	F	32	20	<20	160	320	320	320	80	40	40	40
S98	F	33	160	<20	80	160	320	160	80	20	40	160
S99	F	63	40	<20	20	40	20	20	40	40	20	40
S100	M	27	160	<20	320	160	160	40	80	40	<20	20
S103	F	29	80	80	<20	<20	<20	<20	<20	<20	<20	<20
S104	F	53	160	<20	80	160	80	40	40	<20	80	40
S112	M	51	320	640	320	640	160	160	640	160	160	80
S113	M	37	320	<20	40	40	40	<20	20	<20	<20	<20
S119	F	43	80	<20	<20	40	<20	40	20	<20	<20	<20
S131	F	24	20	<20	20	80	20	<20	160	80	<20	<20
S132	M	27	320	80	20	<20	20	20	<20	<20	<20	<20

While several studies have investigated ZIKV neutralizing antibodies^39,41,42,45,46,48^, these studies have almost only been undertaken in the context of active ZIKV infection, and we believe this is the first study to determine the presence of ZIKV neutralizing antibodies in normal, healthy Thais. Interpretation of the results is somewhat contentious in light of the lack of a clearly defined correlation between absolute PRNT values and protective immunity. However, several studies have shown that a PRNT_50_ ≥ 10 is protective for Japanese encephalitis virus both in human^50–52^ and animal^53^ studies. Similarly, a PRNT_50_ ≥ 10 is defined as protective for yellow fever virus^54^. For DENV, the situation is more complex. While some studies report no correlation between PRNT and protective immunity^55^, a second study assessed PRNT_50_ > 11–16 being protective for DENV 1 and 4, but much higher PRNT values are required for protection against DENV 2^56^.

Overall, this study has shown that some 70% of serum samples examined had neutralizing antibodies against ZIKV (at PRNT_50_ ≥ 10), a level that is protective for JEV^50–52^ and YFV^54^. Modeling studies with DENV have suggested that population protection through herd immunity requires about 80% of the population to carry neutralizing antibodies^57^. While the figure of 70% seen here is lower than the predicted 80% figure, it is likely that such a level will significantly dampen the transmissibility of ZIKV, and possibly explains why, despite cases of ZIKV being detected over much of Thailand^17^, the outbreaks are typically of small clusters that quickly die out.

## Electronic supplementary material

Supplementary Tables

Raw PRNT data

## Data Availability

All data generated or analysed during this study are included in this published article (and its Supplementary Information files).
