# Supplementary material for: Analysis of Zika virus neutralizing antibodies in normal healthy Thais
Source: Sci Rep. 2018 Nov 21;8:17193. doi: 10.1038/s41598-018-35643-6 (PMC6249253; doi:10.1038/s41598-018-35643-6)
Supplement: Supplementary file 1 — Supplementary Tables [file 41598_2018_35643_MOESM1_ESM.pdf]

Analysis of Zika virus neutralizing antibodies in normal healthy Thais

Wannapa Sornjai[a], Janejira Jaratsittisin[b], Prasert Auewarakul[a], Nitwara Wikan[b]* and Duncan R. Smith[b]*

[a]Department of Microbiology, Faculty of Medicine, Siriraj Hospital, Mahidol University, Thailand.

[b]Institute of Molecular Biosciences, Mahidol University, Thailand

*Correspondence to
Duncan R. Smith (duncan_r_smith@hotmail.com) or Nitwara Wikan (nitwara.wik@mahidol.ac.th)
Institute of Molecular Biosciences
Mahidol University, Salaya Campus
25/25 Phuttamontol Sai 4, Salaya
Nakhon Pathom
Thailand 73170
Tel (662) 800 3624-8; Fax (662) 4419906

Supplemental Tables S1 to S11 and supplemental references

Supplemental Table S1. Viruses used and their GenBank accession number

Virus	Virus Designation	Strain	GenBank Accession number
Zika virus SV0010/15	ZIKV SV0010	SV0010/15	KX051562.1
Japanese encephalitis virus	JEV	Beijing-1	U70389.1
Laboratory adapted dengue virus serotype 1	DENV 1 lab	16007	AF180818.1
Clinical isolated dengue virus serotype 1	DENV 1 DF	DENV-1/THAI/NS1-114/2006	KM519585
Laboratory adapted dengue virus serotype 2	DENV 2 lab	16681	M84727.1
Clinical isolated dengue virus serotype 2	DENV 2 DF	DENV-2/THAI/NS1-038/2006	KM519586
Laboratory adapted dengue virus serotype 3	DENV 3 lab	16562	KM519588
Clinical isolated dengue virus serotype 3	DENV 3 DF	DENV-3/THAI/NS1-007/2006	-[a]
Laboratory adapted dengue virus serotype 4	DENV 4 lab	1036	KM519590
Clinical isolated dengue virus serotype 4	DENV 4 DF	DENV-4/THAI/NS1-058/2006	KM519591

[a] No GenBank accession number is available (as reported in [Rungruengphol et al., 2015]).

Supplemental Table S2. Summary of recent studies determining ZIKV PRNT and their starting dilutions and percent neutralization definition.

Title of study	Initial dilution (fold)	Positive % neutralization	Nomenclature	Reference
Imported arboviral infections in Italy, July 2014-October 2015: a National Reference Laboratory report.	10	50	PRNT50≥10	[Fortuna et al., 2017]
Development of a Rapid Diagnostic Test Kit to Detect IgG/IgM Antibody against Zika Virus Using Monoclonal Antibodies to the Envelope and Non-structural Protein 1 of the Virus.	10	50	PRNT50≥10	[Kim et al., 2018]
Mosquito Exposure and Chikungunya and Dengue Infection Among Travelers During the Chikungunya Outbreak in the Americas.	10	50	PRNT50≥10	[Lindholm et al., 2017]
A Rapid Zika Diagnostic Assay to Measure Neutralizing Antibodies in Patients.	10	90	PRNT90≥10	[Shan et al., 2017]
Development of Zika Virus Serological Testing Strategies in New York State.	10	90	PRNT90≥10	[Lee et al., 2018]
Development and evaluation of a novel high-throughput image-based fluorescent neutralization test for detection of Zika virus infection.	20	90	PRNT90≥20	[Koishi et al., 2018]
Evaluation of Euroimmun Anti-Zika Virus IgM and IgG Enzyme-Linked Immunosorbent Assays for Zika Virus Serologic Testing.	20	90	PRNT90≥20	[L'Huillier et al., 2017]

Supplemental Table S3. ZIKV neutralizing antibodies at $PRNT_{50} \geq 10$

$PRNT_{50}$ of ZIKV strain SV0010/15 in 135 human serum samples (S1-S136). $PRNT_{50} \geq 10$ was defined as positive.

Sample	$PRNT_{50}$	Sample	$PRNT_{50}$	Sample	$PRNT_{50}$	Sample	$PRNT_{50}$
S1	10	S35	<10	S70	≥20	S104	≥20
S2	≥20	S36	≥20	S71	≥20	S105	10
S3	<10	S37	≥20	S72	≥20	S106	≥20
S4	≥20	S38	≥20	S73	≥20	S107	<10
S5	10	S39	≥20	S74	10	S108	≥20
S6	10	S40	≥20	S75	≥20	S109	≥20
S7	≥20	S41	≥20	S76	≥20	S110	≥20
S8	≥20	S42	≥20	S77	10	S111	≥20
S9	≥20	S43	≥20	S78	<10	S112	≥20
S10	<10	S44	<10	S79	<10	S113	≥20
S11	<10	S45	≥20	S80	≥20	S114	10
S12	<10	S46	10	S81	≥20	S115	<10
S13	<10	S47	10	S82	≥20	S116	≥20
S14	≥20	S48	≥20	S83	≥20	S117	<10
S15	<10	S49	10	S84	≥20	S118	10
S16	<10	S50	≥20	S85	<10	S119	≥20
S17	<10	S51	10	S86	≥20	S120	<10
S18	≥20	S52	10	S87	≥20	S121	≥20
S19	<10	S53	10	S88	<10	S122	<10
S20	10	S54	≥20	S89	≥20	S123	≥20
S21	≥20	S55	≥20	S90	≥20	S124	<10
S22	10	S56	<10	S91	<10	S125	<10
S23	10	S57	≥20	S92	≥20	S126	<10
S24	≥20	S58	<10	S93	<10	S127	≥20
S25	≥20	S59	<10	S94	≥20	S128	≥20
S26	10	S60	10	S95	≥20	S129	<10
S27	10	S61	≥20	S96	<10	S130	<10
S28	<10	S62	≥20	S97	≥20	S131	≥20
S29	<10	S64	<10	S98	≥20	S132	≥20
S30	≥20	S65	<10	S99	≥20	S133	<10
S31	≥20	S66	<10	S100	≥20	S134	≥20
S32	10	S67	<10	S101	≥20	S135	<10
S33	≥20	S68	≥20	S102	10	S136	≥20
S34	<10	S69	≥20	S103	≥20		

Supplemental Table S4. ZIKV neutralizing antibodies at $PRNT_{90} \geq 20$.

$PRNT_{90}$ of ZIKV strain SV0010/15 in 135 human serum samples (S1-S136). $PRNT_{90} \geq 20$ was defined as positive.

Sample	$PRNT_{90}$	Sample	$PRNT_{90}$	Sample	$PRNT_{90}$	Sample	$PRNT_{90}$
S1	<20	S35	<20	S70	40	S104	160
S2	<20	S36	80	S71	<20	S105	<20
S3	<20	S37	<20	S72	<20	S106	<20
S4	<20	S38	<20	S73	<20	S107	<20
S5	<20	S39	320	S74	<20	S108	<20
S6	<20	S40	160	S75	80	S109	<20
S7	<20	S41	160	S76	160	S110	<20
S8	<20	S42	<20	S77	<20	S111	<20
S9	160	S43	20	S78	<20	S112	320
S10	<20	S44	<20	S79	<20	S113	320
S11	<20	S45	<20	S80	<20	S114	<20
S12	<20	S46	<20	S81	320	S115	<20
S13	<20	S47	<20	S82	80	S116	<20
S14	<20	S48	<20	S83	<20	S117	<20
S15	<20	S49	<20	S84	20	S118	<20
S16	<20	S50	640	S85	<20	S119	80
S17	<20	S51	<20	S86	80	S120	<20
S18	<20	S52	<20	S87	<20	S121	<20
S19	<20	S53	<20	S88	<20	S122	<20
S20	<20	S54	<20	S89	160	S123	<20
S21	<20	S55	<20	S90	<20	S124	<20
S22	<20	S56	<20	S91	<20	S125	<20
S23	<20	S57	640	S92	20	S126	<20
S24	<20	S58	<20	S93	<20	S127	<20
S25	<20	S59	<20	S94	<20	S128	<20
S26	<20	S60	<20	S95	<20	S129	<20
S27	<20	S61	<20	S96	<20	S130	<20
S28	<20	S62	160	S97	<20	S131	20
S29	<20	S64	<20	S98	160	S132	320
S30	<20	S65	<20	S99	40	S133	<20
S31	<20	S66	<20	S100	160	S134	<20
S32	<20	S67	<20	S101	<20	S135	<20
S33	1,280	S68	160	S102	<20	S136	<20
S34	<20	S69	<20	S103	80		

Supplemental Table S5. ZIKV neutralizing antibodies at $PRNT_{50} \geq 20$

$PRNT_{50}$ of ZIKV strain SV0010/15 in 135 human serum samples (S1-S136). $PRNT_{50} \geq 20$* was defined as positive

Sample	$PRNT_{50}$	Sample	$PRNT_{50}$	Sample	$PRNT_{50}$	Sample	$PRNT_{50}$
S1	<20	S35	<20	S70	160	S104	>320
S2	20	S36	>320	S71	40	S105	<20
S3	<20	S37	80	S72	40	S106	80
S4	20	S38	80	S73	<20	S107	<20
S5	<20	S39	>1,280	S74	<20	S108	<20
S6	20	S40	>320	S75	>320	S109	<20
S7	20	S41	>320	S76	>320	S110	<20
S8	20	S42	80	S77	<20	S111	<20
S9	640	S43	>320	S78	<20	S112	1,280
S10	<20	S44	20	S79	<20	S113	1,280
S11	<20	S45	40	S80	20	S114	20
S12	<20	S46	<20	S81	1,280	S115	<20
S13	<20	S47	<20	S82	320	S116	80
S14	20	S48	80	S83	80	S117	<20
S15	<20	S49	<20	S84	160	S118	20
S16	<20	S50	2,560	S85	<20	S119	160
S17	<20	S51	<20	S86	>320	S120	<20
S18	20	S52	<20	S87	40	S121	40
S19	<20	S53	<20	S88	<20	S122	<20
S20	80	S54	80	S89	>320	S123	40
S21	80	S55	40	S90	20	S124	<20
S22	40	S56	<20	S91	<20	S125	<20
S23	40	S57	>1,280	S92	40	S126	<20
S24	40	S58	<20	S93	<20	S127	160
S25	40	S59	<20	S94	<20	S128	20
S26	<20	S60	<20	S95	40	S129	<20
S27	20	S61	160	S96	<20	S130	<20
S28	<20	S62	>320	S97	20	S131	160
S29	<20	S64	<20	S98	>320	S132	2,560
S30	20	S65	<20	S99	160	S133	<20
S31	40	S66	<20	S100	>320	S134	40
S32	20	S67	<20	S101	<20	S135	<20
S33	>1,280	S68	>320	S102	<20	S136	80
S34	<20	S69	80	S103	>320		

* Samples were not titrated to limit of neutralization.

Supplemental Table S6. Gender of donors of samples screened for DENV and JEV PRNT by ZIKV PRNT status

Sample group	Gender		Total
	Male	Female	
ZIKV $PRNT_{90} < 20$	7	23	30
ZIKV $PRNT_{90} \geq 20$	7	23	30

Supplemental Table S7. Age of donors of samples screened for DENV and JEV PRNT by ZIKV PRNT status

Age range	Sample group	
	ZIKV $PRNT_{90} < 20$	ZIKV $PRNT_{90} \geq 20$
18-30	12	10
31-40	8	9
41-50	5	4
≥51	5	7
Total	30	30

Supplemental Table S8. Full screen of neutralizing antibody titer for ZIKV DENV (1-4) and JEV by plaque reduction neutralization test for two control samples ($PRNT_{90} \leq 20$).

Sample	Gender	Age	ZIKV SV0010/15	JEV	DENV 1 lab	DENV 1 DF	DENV 2 lab	DENV 2 DF	DENV 3 lab	DENV 3 DF	DENV 4 lab	DENV 4 DF
S137	M	57	-	-	-	-	-	-	-	-	-	-
S63	F	22	1,280	640	160	160	320	160	160	20	160	320

Supplemental Table S9. Full screen of neutralizing antibody titer for DENV (1-4) and JEV by plaque reduction neutralization test of ZIKV $PRNT_{90} \geq 20$ negative serum samples*.

Sample	Gender	Age	ZIKV SV0010/15	JEV	DENV 1 lab	DENV 1 DF	DENV 2 lab	DENV 2 DF	DENV 3 lab	DENV 3 DF	DENV 4 lab	DENV 4 DF
S1	F	42	< 20	80	40	20	160	160	40	20	< 20	< 20
S2	F	29	< 20	< 20	320	320	20	20	20	< 20	20	20
S3	F	27	< 20	80	< 20	< 20	< 20	< 20	< 20	< 20	< 20	< 20
S4	F	32	< 20	20	160	160	< 20	< 20	< 20	< 20	< 20	< 20
S5	F	25	< 20	80	< 20	< 20	< 20	< 20	< 20	< 20	< 20	< 20
S6	M	42	< 20	< 20	80	80	320	160	80	20	20	20
S7	F	25	< 20	< 20	320	160	320	160	160	80	20	80
S8	F	29	< 20	20	80	40	320	160	80	80	< 20	< 20
S10	M	30	< 20	< 20	20	20	20	< 20	80	40	< 20	< 20
S11	F	27	< 20	40	320	640	40	40	40	20	< 20	< 20
S12	F	26	< 20	40	< 20	< 20	< 20	< 20	< 20	< 20	< 20	< 20
S13	F	28	< 20	< 20	< 20	< 20	< 20	< 20	< 20	< 20	< 20	< 20
S14	F	28	< 20	< 20	< 20	< 20	320	160	< 20	< 20	20	20
S15	F	35	< 20	< 20	< 20	20	< 20	< 20	80	40	< 20	< 20
S60	F	35	< 20	20	80	80	320	160	40	20	< 20	40
S64	F	36	< 20	< 20	< 20	< 20	80	160	< 20	< 20	40	40
S67	M	37	< 20	< 20	< 20	< 20	< 20	< 20	< 20	< 20	< 20	< 20
S77	F	52	< 20	< 20	40	160	20	< 20	20	< 20	40	40
S79	M	34	< 20	20	40	160	80	80	320	320	80	40
S91	M	27	< 20	40	< 20	< 20	< 20	< 20	< 20	< 20	< 20	< 20
S96	F	44	< 20	< 20	160	160	80	160	40	20	80	80
S101	M	20	< 20	< 20	40	80	< 20	< 20	640	320	< 20	20
S114	M	37	< 20	20	20	40	80	160	< 20	20	< 20	20
S118	F	52	< 20	< 20	160	160	80	80	40	40	40	40
S120	F	41	< 20	< 20	< 20	< 20	< 20	< 20	< 20	< 20	< 20	< 20
S122	F	47	< 20	< 20	40	160	80	20	40	20	40	160
S123	F	54	< 20	< 20	320	640	160	80	160	160	20	20
S125	F	52	< 20	< 20	20	40	40	20	320	160	< 20	20
S127	F	42	< 20	20	160	160	320	160	40	20	20	40
S133	F	51	< 20	20	< 20	< 20	< 20	< 20	< 20	< 20	< 20	< 20

*Plaque reduction neutralization test of selected flaviviruses in 30 serum samples that showed ZIKV $PRNT_{90} < 20$. The PRNT cutoff value at 90 ($PRNT_{90}$) was defined as a reciprocal of the highest dilution of tested serum that resulted in the reduction of viral infectivity by 90%.

Supplemental Table S10. ZIKV PRNT90 positive and negative samples in relationship to other flavivirus PRNT results*.

Sample group	Number of flavivirus $PRNT_{90} \geq 20$						Total
	0	1	2	3	4	5	
ZIKV $PRNT_{90} < 20$	3	5	4	2	12	4	30
ZIKV $PRNT_{90} \geq 20$	2	2	0	8	9	9	30

* PRNTs for DENV 1-4 are counted separately

Supplemental Table S11. ZIKV $PRNT_{90}$ positive and negative samples in relationship to other flavivirus PRNT results*.

Sample group	Number of flavivirus $PRNT_{90} \geq 20$			Total
	0	1	2	
ZIKV $PRNT_{90} < 20$	3	19	8	30
ZIKV $PRNT_{90} \geq 20$	2	16	12	30

* $PRNT_{90} \geq 20$ for any DENV serotype is counted as positive for DENV

Supplemental References

Fortuna C, Remoli ME, Rizzo C, Benedetti E, Fiorentini C, Bella A, Argentini C, Farchi F, Castilletti C, Capobianchi MR, Zammarchi L, Bartoloni A, Zanchetta N, Gismondo MR, Nelli LC, Vitale G, Baldelli F, D'Agaro P, Sodano G, Rezza G, Arbovirus Working G, Venturi G. (2017). Imported arboviral infections in Italy, July 2014-October 2015: a National Reference Laboratory report. BMC Infect Dis, 17:216.

Kim YH, Lee J, Kim YE, Chong CK, Pinchemel Y, Reisdorfer F, Coelho JB, Dias RF, Bae PK, Gusmao ZPM, Ahn HJ, Nam HW. (2018). Development of a Rapid Diagnostic Test Kit to Detect IgG/IgM Antibody against Zika Virus Using Monoclonal Antibodies to the Envelope and Non-structural Protein 1 of the Virus. Korean J Parasitol, 56:61-70.

Koishi AC, Suzukawa AA, Zanluca C, Camacho DE, Comach G, Duarte Dos Santos CN. (2018). Development and evaluation of a novel high-throughput image-based fluorescent neutralization test for detection of Zika virus infection. PLoS Negl Trop Dis, 12:e0006342.

L'Huillier AG, Hamid-Allie A, Kristjanson E, Papageorgiou L, Hung S, Wong CF, Stein DR, Olsha R, Goneau LW, Dimitrova K, Drebot M, Safronetz D, Gubbay JB. (2017). Evaluation of Euroimmun Anti-Zika Virus IgM and IgG Enzyme-Linked Immunosorbent Assays for Zika Virus Serologic Testing. J Clin Microbiol, 55:2462-2471.

Lee WT, Wong SJ, Kulas KE, Dupuis AP, 2nd, Payne AF, Kramer LD, Dean AB, St George K, White JL, Sommer JN, Ledizet M, Limberger RJ. (2018). Development of Zika Virus Serological Testing Strategies in New York State. J Clin Microbiol, 56.

Lindholm DA, Myers T, Widjaja S, Grant EM, Telu K, Lalani T, Fraser J, Fairchok M, Ganesan A, Johnson MD, Kunz A, Tribble DR, Yun HC. (2017). Mosquito Exposure and Chikungunya and Dengue Infection Among Travelers During the Chikungunya Outbreak in the Americas. Am J Trop Med Hyg, 96:903-912.

Rungruengphol C, Jaresitthikunchai J, Wikan N, Phaonakrop N, Keadsanti S, Yoksan S, Roytrakul S, Smith DR. (2015). Evidence of plasticity in the dengue virus: Host cell interaction. Microb Pathog, 86:18-25.

Shan C, Ortiz DA, Yang Y, Wong SJ, Kramer LD, Shi PY, Loeffelholz MJ, Ren P. (2017). Evaluation of a Novel Reporter Virus Neutralization Test for Serological Diagnosis of Zika and Dengue Virus Infection. J Clin Microbiol, 55:3028-3036.
